# Chromosomal Location and Comparative Genomics Analysis of Powdery Mildew Resistance Gene *Pm51* in a Putative Wheat-*Thinopyrum ponticum* Introgression Line

**DOI:** 10.1371/journal.pone.0113455

**Published:** 2014-11-21

**Authors:** Haixian Zhan, Guangrong Li, Xiaojun Zhang, Xin Li, Huijuan Guo, Wenping Gong, Juqing Jia, Linyi Qiao, Yongkang Ren, Zujun Yang, Zhijian Chang

**Affiliations:** 1 School of Life Science and Technology, University of Electronic Science and Technology of China, Chengdu, Sichuan, China; 2 Crop Science Institute, Shanxi Academy of Agricultural Sciences, Taiyuan, Shanxi, China; 3 Key Lab of Crop Gene Resources and Germplasm Enhancement on Loess Plateau, Ministry of Agriculture, Taiyuan, Shanxi, China; 4 College of agronomy, Shanxi Agricultural University, Taigu, Shanxi, China; Morehouse School of Medicine, United States of America

## Abstract

Powdery mildew (PM) is a very destructive disease of wheat (*Triticum aestivum* L.). Wheat-*Thinopyrum ponticum* introgression line CH7086 was shown to possess powdery mildew resistance possibly originating from *Th. ponticum*. Genomic *in situ* hybridization and molecular characterization of the alien introgression failed to identify alien chromatin. To study the genetics of resistance, CH7086 was crossed with susceptible genotypes. Segregation in F_2_ populations and F_2:3_ lines tested with Chinese *Bgt* race E09 under controlled conditions indicated that CH7086 carries a single dominant gene for powdery mildew resistance. Fourteen SSR and EST-PCR markers linked with the locus were identified. The genetic distances between the locus and the two flanking markers were 1.5 and 3.2 cM, respectively. Based on the locations of the markers by nullisomic-tetrasomic and deletion lines of ‘Chinese Spring’, the resistance gene was located in deletion bin 2BL-0.89-1.00. Conserved orthologous marker analysis indicated that the genomic region flanking the resistance gene has a high level of collinearity to that of rice chromosome 4 and *Brachypodium* chromosome 5. Both resistance specificities and tests of allelism suggested the resistance gene in CH7086 was different from previously reported powdery mildew resistance genes on 2BL, and the gene was provisionally designated *PmCH86*. Molecular analysis of *PmCH86* compared with other genes for resistance to *Bgt* in the 2BL-0.89-1.00 region suggested that *PmCH86* may be a new PM resistance gene, and it was therefore designated as *Pm51*. The closely linked flanking markers could be useful in exploiting this putative wheat-*Thinopyrum* translocation line for rapid transfer of *Pm51* to wheat breeding programs.

## Introduction

Powdery mildew (PM), caused by *Blumeria graminis* f. sp. *tritici* (*Bgt*), is a globally important disease of wheat (*Triticum aestivum* L.). Resistant varieties are the most feasible means of controlling the disease and reducing yield losses. To date, 54 formally designated *Pm* resistance genes have been identified. They have been mapped to 46 loci and assigned to specific chromosomes or chromosome arms [1]. Of these loci, 29 genes were transferred from relatives, including *T. turgidum* var. *dicoccoides*, *T. timopheevii*, *T. monococcum*, *Aegilops tauschii*, *Ae. speltoides*, *Ae. longissima*, *Ae. ovata*, and from more distantly related species, including *Secale cereale*, *Dasypyrum villosum*, *and Thinopyrum intermedium* [2]. However, many resistance genes become ineffective because of frequent changes in pathogen populations, especially when a single resistance gene is deployed over a wide area. Therefore, new sources of effective and durable resistance from both common wheat and wild relatives are required for resistance breeding.

Tall wheatgrass, *Thinopyrum ponticum* (Podp.) Z.-W. Liu & R.-C. Wang [syn. *Agropyron elongatum* (Host) Beauv., *Lophopyrum ponticum* (Podp.) A. Löve, and *Elytrigia elongata* (Host) Nevski], has been one of the most beneficial perennial species that conferred valuable genetic variability for wheat improvement. In addition to wheat rust resistances, *Th. ponticum* displays resistance to powdery mildew, eyespot, wheat streak mosaic virus (WSMV), wheat curl mite (WCM), Cephalosporium stripe, and Fusarium head blight - [3]. As for rust resistance transferred from *Th. ponticum*, three genes for resistance to leaf rust viz. *Lr19*, *Lr24*, and *Lr29*, and three genes for resistance to stem rust, viz. *Sr24, Sr25*, and *Sr26*, were reported in wheat-*Th. ponticum* translocation derivatives [4],[5]. However, there are few reports on the transfer of powdery mildew resistance from *Th. ponticum* to wheat [3].

So called cryptic translocations between wheat and alien chromatin have been reported on a number of occasions. These are alien transfers that cannot be visualized by cytological means, and are also usually not detectable with markers. Recent examples were the transfers of rust resistances from *Ae. geniculata* and *Ae. triuncialis* to wheat [6],[7]. Genomic rearrangements in wheat hybrids due to cryptic introgressions of small chromosome segments from *Dasypyrum villosum* and *Th. ponticum* to wheat were also reported recently [8],[9]. However, with materials having putative cryptic translocations that cannot be detected cytologically or with markers there is always the question of alien identity.

CH7086, a *Th. ponticum*-derived wheat introgression line, was resistant to powdery mildew under greenhouse conditions in Taiyuan, Shanxi province. The resistance gene was preliminarily assigned to chromosome arm 2BL [10]. The objectives of the present study were to characterize this potential new cryptic wheat-*Th. ponticum* translocation, and to determine its location using microsatellite and comparative genomic molecular marker analyses.

## Materials and Methods

### Plant materials and populations

The materials used in this study were *Th. ponticum* (accession R431) with the genomic formula JJJJ^s^J^s^ [11]; partial amphiploid, Xiaoyan 7430, derived from accession R431 and provided by the Crop Science Institute, Shanxi Academy of Agricultural Sciences, Taiyuan; wheat genotypes ‘CH7086’, ‘CH5241’, ‘Zhong 8701’, ‘Jimai 26’, ‘Xiangyang 4’, ‘Misuizao’, and ‘Chinese Spring (CS)’; and various ‘CS’ nullisomic-tetrasomic (NT) stocks and deletion lines, obtained from Dr. B. Friebe, Wheat Genetic and Genomic Resources Center, Kansas State University, USA. CH7086 and CH5241 are homogeneous BC_2_F_5_-derived wheat lines obtained from the cross Zhong 8701/Xiaoyan 7430//2*Jimai 26. CH7086 is resistant to powdery mildew whereas CH5241 is susceptible. Xiaoyan 7430, the resistance donor of CH7086, was derived from the cross Misuizao/R431//Xiangyang 4 [12].

To investigate the inheritance of powdery mildew resistance introgressed from *Th. ponticum*, CH7086 was crossed to susceptible cultivars CH5241, Taichung 29, SY95-71, and Jintai 170 to generate segregating populations. The F_2_ and F_3_ were tested for segregation of powdery mildew response. An F_2_ population of 154 plants and 148 derived F_3_ lines from CH7086/CH5241 were used for microsatellite screening and gene mapping. CH7086, Xiaoyan 7430, the original mildew resistant donor accession (R431) of *Th. ponticum*, and CS were also used for genomic in situ hybridization and C-banding analyses to determine the chromosomal composition in Xiaoyan 7430 and the size of any alien introgressions in CH7086.

### Genomic *in situ* hybridization

Seedling root tips were collected, pretreated in ice water for 24 h and fixed in ethanol-acetic acid (3∶1) for one week. Root-tip squashes and the conventional Giemsa-C banding methods were performed according to Gill et al. [13]. For GISH analysis, total genomic DNA from *Th. ponticum* was labeled with fluorescein-12-dUTP by nick translation following the manufacturer's instructions (Roche Diagnostics, Indianapolis, IN). Sheared genomic DNA of Chinese Spring wheat (AABBDD, 2*n* = 6*x* = 42) was used as blocking DNA, and the probe-to-blocker ratio was approximately 1∶120. The detection and visualization of GISH signals were performed as described by Han et al. [14]. Images of GISH and C-banded chromosomes were taken with an Olympus BX-51 microscope using a DP70 CCD camera (Olympus, Japan).

### Testing for powdery mildew response

The seedling reactions of line CH7086 and the parental lines inoculated with four *Bgt* isolates, provided by the Plant Protection Institute, Chinese Academy of Agricultural Sciences, are shown in Table 1. Among the *Bgt* isolates tested, E09, a prevalent pathotype in the Beijing area, is virulent to *Pm1*, *Pm3a*, *Pm3c*, *Pm3e*, *Pm5a*, *Pm6*, *Pm7*, *Pm8*, *Pm17*, and *Pm19* [15]; E20 and E21 are the most widely virulent pathotypes in China and are virulent to most of the *Pm* genes including *Pm4a*, *Pm4b*, *PmPs5A*, and *Pm33*. E26 is virulent to *Pm4b* and *Pm33*, but avirulent to *Pm4a* and *PmPs5A* [16],[17]; E15, avirulent to*Pm6*, was used for the test of allelism between *Pm6* and the resistance gene in CH7086 [16]. Procedures used in powdery mildew inoculation, incubation of inoculated plants, and reaction scoring were as described in He et al. [18]. All F_2_ plants and their parents were inoculated with isolate E09 at the one-leaf seedling stage to screen for powdery mildew reaction. For progeny testing, 15 to 20 F_3_ seedlings from each F_2_ plant were grown and tested with the same race. The infection types (ITs) were rated on a 0–4 scale, 7–10 days after inoculation when conidia were fully developed [18].

**Table 1:** Reactions of selected donor materials, parents and controls after with four *Bgt* isolates.

Line	Chromosome	Genomic	*Bgt* isolate			
	number	formula	E09	E20	E21	E26
*Th. ponticum* R431	70	JJJJ^s^J^s^	0	0	0	0
Xiaoyan (XY) 7430	56	ABD +JJ^s^	0	0;	0;	0
Xiangyang 4	42	ABD	4	3	3	4
Misuizao	42	ABD	4	3	4	3
CH7086	42	ABD	0	0;	0	0;
CH5241	42	ABD	4	4	4	3
Zhong 8701	42	ABD	4	4	4	4
Jimai 26	42	ABD	4	3	4	4
SY95-71	42	ABD	4	4	4	4
Jintai 170	42	ABD	4	4	4	3
Taichung 29	42	ABD	4	4	4	4

Infection types were based on a 0–4 scale, where 0 =  no visible symptoms, 0;  =  necrotic flecks, 1 =  necrosis with low sporulation, 2 =  necrosis with moderate sporulation, 3 =  no necrosis with moderate to high sporulation, and 4 =  no necrosis with full sporulation. Scores of 0–2 were classified as resistant and 3–4 as susceptible.

### Molecular marker analysis

Wheat chromosome 2BL has good synteny with rice chromosome 4 [19],[20]. Thus, wheat ESTs that mapped to bin 2BL- 0.50–1.00 were aligned to the rice genome sequences using BLASTN. ESTs with orthologous genes in the syntenic region of rice chromosome 4 were used to develop STS (sequence tagged site) markers. PCR products from STS primers were separated in 1% agarose gels, whereas PCR products from EST and SSR primers were separated in 8% non-denaturing polyacrylamide gels.

In order to produce SCAR markers, amplified polymorphic bands between CH7086 and Taichung 29 were extracted from gels and re-amplified. The method of cloning and sequencing PCR products was as described by Liu et al. [21].

### Chromosome assignment and linkage analysis

Chi-squared (χ^2^) tests for goodness-of-fit were used to test for deviations of observed data from theoretically expected segregations. Linkages between DNA markers and the resistance gene were established with JoinMap version 4.0 software (Wageningen, Netherlands) with a LOD threshold of 3.0. Map distances were determined using the Kosambi mapping function.

## Results

### Likely origin of the powdery mildew resistance

Seedling reactions of *Th. ponticum*, the partial amphiploid donor and nine wheat cultivars/lines to four *Bgt* isolates are summarized in Table 1. CH7086 and the *Th. ponticum* parent R431 were resistant to isolates E09, E20, E21, and E26 (IT 0-0;), whereas the wheat parents or lines, Xiangyang 4, Misuizao, Zhong 8701, and Jimai 26, were susceptible (IT 3-4). These results demonstrated that CH7086 was resistant to powdery mildew, with the ITs being similar to the donor Xiaoyan 7430 (IT 0-0;) as well as the donor *Th. ponticum* accession R431 (IT 0).

### Attempted characterization of an alien introgression in CH7086

**Figure 1:**
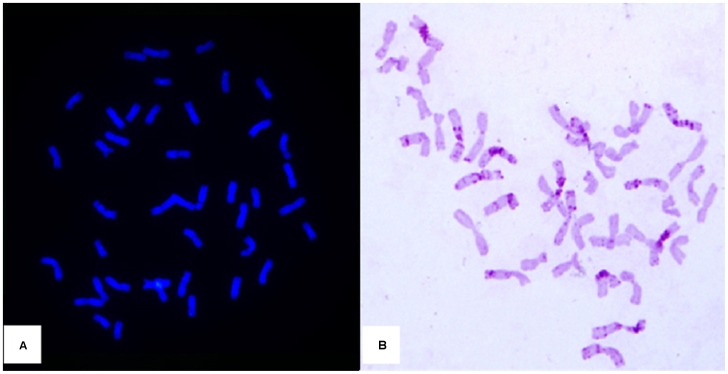
Cytogenetic analysis of CH7086. A) GISH patterns of CH7086 using *Th. ponticum* genomic DNA (labeled with fluorescein-12-dUTP) as probe. Cell was counterstained with DAPI and fluoresces blue. No GISH signal was observed in CH7086. B) Giemsa-C banding of CH7086.

When GISH using *Th. ponticum* genomic DNA as probe was performed on the wheat-*Th. ponticum* partial amphiploid Xiaoyan 7430 (2*n* = 56) and line CH7086, 14 *Th. ponticum* chromosomes were clearly distinguishable in the mitotic metaphases of Xiaoyan 7034 (results not shown). However, no GISH signals were observed in CH7086 (Figure 1A). Giemsa-C banding (Figure 1B) indicated that CH7086 contained typical wheat chromosomes without visible bands indicative of *Thinopyrum* chromatin.

### Genetic analysis of powdery mildew resistance in CH7086

Wheat-*Th. ponticum* introgression line CH7086, the F_1_ hybrid, and F_2:3_ families from CH7086 crossed respectively with wheat lines Taichung 29, CH5241, SY95-71, and Jintai 170 were inoculated with *Bgt* isolate E09. CH7086 was highly resistant (IT 0) and the wheat lines were all highly susceptible (IT 4) (Table 1). All F_1_ hybrid seedlings and adult plants were highly resistant, implying that the resistance in CH7086 was dominant. F_2_ populations from four crosses segregated 3 resistant: 1 susceptible and the pooled data of F_2:3_ lines from CH5241/CH7086 and CH7086/Taichung 29 segregated 63 homozygous resistant: 128 segregating: 61 homozygous susceptible, as expected for single gene segregation (χ^2^_1:2:1_ = 0.10, *P*_df2_ = 0.95) (Table 2). The dominant gene for *Pm* resistance in CH7086 was temporarily designated as *PmCH86*.

**Table 2:** Mildew reactions and segregation ratios of F_2_ plants and derived F_3_ lines following inoculation with Bgt isolate E09.

Parent or cross		No. of plants or lines			Expected	?^2^	*P*
		Resistant	Segregating	Susceptible	ratio		
CH7086	P_1_	19					
SY95-71	P_2_			15			
CH5241	P_3_			13			
Taichung 29	P_4_			13			
Jintai 170	P_5_			17			
CH7086/Taichung 29	F_1_	11					
	F_2_	88		26	3∶1	0.292	0.589
	F_2:3_	25	52	27	1∶2∶1	0.077	0.962
CH7086/SY95-71	F_1_	7					
	F_2_	118		42	3∶1	0.133	0.715
CH5241/CH7086	F_1_	21					
	F_2_	111		43	3∶1	0.701	0.402
	F_2:3_	38	76	34	1∶2∶1	0.324	0.850
CH7086/Jintai 170	F_1_	9					
	F_2_	149		40	3∶1	1.483	0.223
Pooled F_2_ data		466		151	3∶1	0.091	0.763
Pooled F_2:3_ data		63	128	61	1∶2∶1	0.095	0.953

Values for significance of *χ* ^2^ at *P* = 0.05, and 3.83 for 1 *df* and 5.99 for 2 *df*, respectively.

### Identification and physical bin mapping of polymorphic markers linked to *PmCH86*

**Figure 2:**
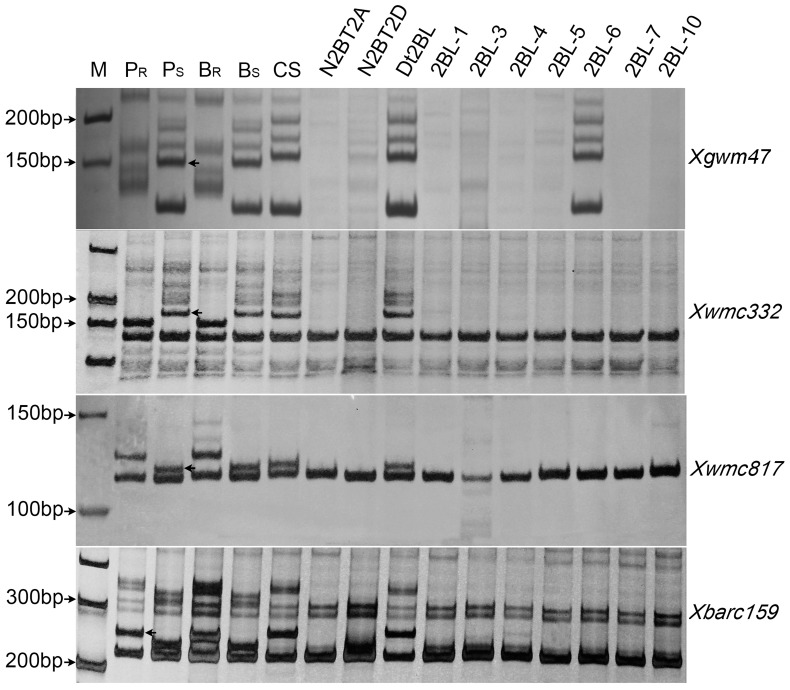
PCR amplification patterns of three linked microsatellite markers. M: DNA ladder; P_R_: CH7086; P_S_: CH5241; B_R_: resistant bulk; B_S_: susceptible bulk; CS: Chinese Spring; N2BT2A and N2BT2D: nullisomic-tetrasomic lines; 2BL-1, 2BL-3, 2BL-4, 2BL-5, 2BL-6, 2BL-7 and 2BL-10: homozygous deletion lines of 2BL of CS; Arrows indicate the critical bands.

The 148 plants of the F_2_ population segregated 1∶2∶1 for all fourteen markers (Table 3, Table 4). Of the microsatellite markers tested, *Xgwm47*, *Xwmc332*, *Xwmc317*, *Xwmc817*,and *Xbarc159* showed linkage with powdery mildew resistance in CH7086. As these markers all map to chromosome arm 2BL, *PmCH86* must also be located in this arm. Based on the high-density microsatellite consensus map of common wheat [22], two microsatellite markers (*Xwmc332* and *Xbarc159*) linked with the resistance in this study were mapped at a distance of 45.7 cM on chromosome arm 2BL. As shown in Figure 2, we bin mapped the five SSR markers by CS deletion lines. *Xgwm47* was the only marker located in bin 0.59–0.89 and the others, *Xwmc332*, *Xwmc317*, *Xwmc817*, and *Xbarc159*, mapped to the 2BL-6 deletion bin FL 0.89–1.00. *PmCH86* was placed in the interval between *Xwmc332* and *Xwmc317*, thus the physical location of *PmCH86* was in the 2BL-6 deletion bin, which is the most distal bin of the long arm accounting for approximately 11% of the physical length of this chromosome arm. Evaluation of the five linked microsatellite markers, including four codominant and one dominant (Table 3, Table 4, Figure 2), along the physical and genetic maps of chromosome 2B allowed us to estimate the genomic location of *PmCH86*.

**Table 3:** Segregations of powdery mildew resistance and markers linked to *PmCH86* in F_2_ population or F_2:3_ lines from CH5241/CH7086.

Marker	Resistance	Codominant marker			Dominant marker				Total	?^2^_(1:2:1or 3:1)_	*P*
	genotype	AA	Aa	aa	AA	Not AA	Not aa	aa			
*Xgwm47*	*PmPm*	24	6	8					38^a^		
	*Pmpm*	9	59	8					76		
	*pmpm*	5	4	25					34		
	Total	38	69	41					148	0.797^b^	0.671
*Cos66*	*PmPm*	31	7	0					38		
	*Pmpm*	8	62	6					76		
	*pmpm*	0	4	30					34		
	Total	39	73	36					148	0.149	0.928
*Xbcd135*	*PmPm*				38	0			38		
	*Pmpm*				73	3			76		
	*pmpm*				4	30			34		
	Total				115	33			148	0.577	0.448
*Cos55*	*PmPm*						34	4	38		
	*Pmpm*						3	73	76		
	*pmpm*						1	33	34		
	Total						38	110	148	0.036	0.849
*Cos65*	*PmPm*	34	3	1					38		
	*Pmpm*	3	72	1					76		
	*pmpm*	1	3	30					34		
	Total	38	78	32					148	0.919	0.632
BI479701	*PmPm*				37	1			38		
	*Pmpm*				75	1			76		
	*pmpm*				4	30			34		
	Total				116	32			148	0.901	0.343
P79	*PmPm*	31	6	1					38		
	*Pmpm*	2	72	2					76		
	*pmpm*	1	2	31					34		
	Total	34	80	34					148	0.973	0.615

AA homozygous for the CH7086 allele; aa homozygous for the CH5241 allele; Aa heterozygous.

*^a^*The data of F_2:3_ lines from CH5241/CH7086;

*^b^*Values for significance of *χ* ^2^ at *P* = 0.05 is 3.84 for 1 *df* and 5.99 for 2 *df*, respectively.

**Table 4:** Segregations of powdery mildew resistance and markers linked to *PmCH86* in F_2_ population or F_2:3_ lines from CH5241/CH7086.

Marker	Resistance	Codominant marker			Dominant marker				Total	?^2^_(1:2:1or 3:1)_	*P*
	genotype	AA	Aa	aa	AA	Not AA	Not aa	aa			
*Xwmc332*	*PmPm*	34	3	1					38		
	*Pmpm*	1	73	2					76		
	*pmpm*	1	2	31					34		
	Total	36	78	34					148	0.486	0.784
BQ246670	*PmPm*	37	1	0					38		
	*Pmpm*	0	75	1					76		
	*pmpm*	1	0	33					34		
	Total	38	76	34					148	0.324	0.850
BE444894	*PmPm*						21	17	38		
	*Pmpm*						8	68	76		
	*pmpm*						3	31	34		
	Total						32	116	148	0.901	0.343
*Xwmc317*	*PmPm*				30	8			38		
	*Pmpm*				65	11			76		
	*pmpm*				12	22			34		
	*PmPm*				107	41			148	0.577	0.448
*Xwmc817*	*PmPm*	19	12	7					38		
	*Pmpm*	19	46	11					76		
	*pmpm*	6	5	23					34		
	Total	44	63	41					148	3.392	0.183
BE405017	*PmPm*						16	22	38		
	*Pmpm*						20	56	76		
	*pmpm*						8	26	34		
	Total						44	104	148	1.766	0.184
*Xbarc159*	*PmPm*	10	17	11					38		
	*Pmpm*	19	42	15					76		
	*pmpm*	8	8	18					34		
	Total	37	67	44					148	1.986	0.370

AA homozygous for the CH7086 allele; aa homozygous for the CH5241 allele; Aa heterozygous.

^*a*^The data of F_2:3_ lines from CH5241/CH7086;

^*b*^Values for significance of *χ* ^2^ at *P* = 0.05 is 3.84 for 1 *df* and 5.99 for 2 *df*, respectively.

### Comparative genomics analysis

**Figure 3:**
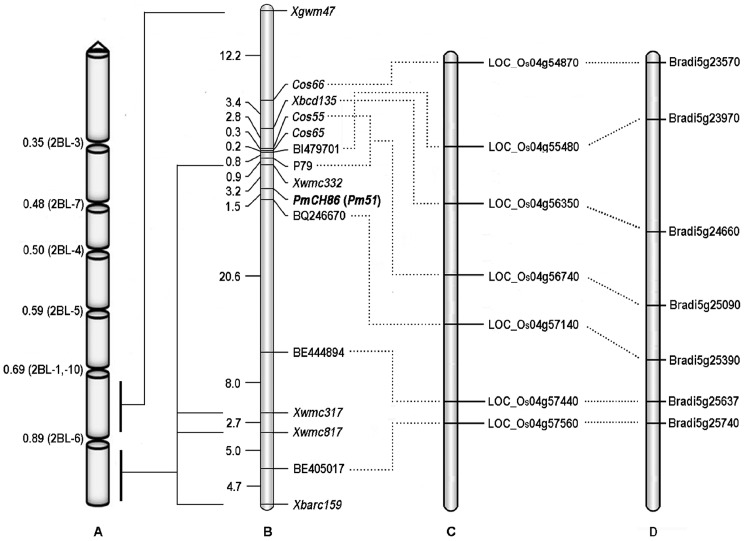
Genetic and comparative mapping of the *PmCH86* gene. A) Chromosome physical map of 2BL (http://www.k-state.edu/wgrc/Germplasm/Deletions/grp2L.html). B) Genetic map of wheat chromosome 2BL, only the region relatively close to *PmCH86* was shown. Genetic distances are shown to the left in cM. C) The homologous region of *PmCH86* with rice chromosome 4 (http://rice.plantbiology.msu.edu/). D) The homologous region of *PmCH86* with *Brachypodium* chromosome 5 (http://www.brachypodium.org/database).

Powdery mildew resistance genes *Pm6*, *Pm33*, and *PmJM22* are also located in the distal region of chromosome 2BL [23],[24],[25]. To clarify the relationship of *PmCH86* with these known genes, we designed the primers for molecular markers based on wheat EST and syntenic regions of the rice. An STS marker *Xbcd135* which co-segregated with *Pm6* was also developed (Table S1). Comparative mapping was reported between wheat chromosome arm 2BL, rice chromosome 4 and *Brachypodium* chromosome 5 [19],[25]. Eleven STS and EST-STS flanking markers, BF478581, DN949092, BM138525, CINAU140, BI479701, BQ169948, BQ169948, BQ246670, BE500840, BE444894, and BE405017, were used as queries to search for orthologous genes in rice and *Brachypodium* genomic sequences. Both Cos66 and BE405017 detected orthologs on the long arm terminal regions of rice chromosome 4 (LOC_Os04g54870 and LOC_Os04g57560) and *Brachypodium* chromosome 5 (Bradi5g23570 and Bradi5g25740). The closest EST markers flanking *PmCH86* were BI479701 and BQ246670 at distances of 4.9 cM and 1.5 cM, respectively; BI479701 was ortholog of rice gene LOC_Os04g55480 and *Brachypodium* gene Bradi5g23970. The ortholog of BQ246670 was found in rice (LOC_Os04g57140) and *Brachypodium* (Bradi5g25390). The terminal region of wheat 2BL had a similar gene order to rice 4L and *Brachypodium* 5L. Thus, the collinear region of the powdery mildew resistance gene *PmCH86* covered 214 kb genomic region (LOC_Os04g56740 to LOC_Os04g57140) of chromosome 4 L in rice and 198 kb genomic region (Bradi5g25090 to Bradi5g25390) of chromosome 5 L in *Brachypodium* (Table S1, Figure 3). The orthologous genomic regions of *PmCH86* in the rice and *Brachypodium* genomes could be candidate regions for fine mapping of *PmCH86*.

### Validation of the flanking markers in marker-assisted selection

**Figure 4:**
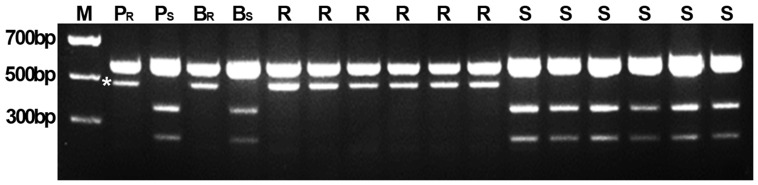
A profile of amplification with marker BQ246670 in F_2_ population from cross of CH7086/Taichung 29. M: DNA ladder; P_R_: CH7086; P_S_: Taichung 29; B_R_: resistant bulk; B_S_: susceptible bulk; R: homozygous resistant F_2_ plants, S: homozygous susceptible F_2_ plants. Asterisk indicates the critical band linked with *PmCH86*.

The closest markers *Xwmc332* and BQ246670 were linked to *PmCH86* with genetic distances of 3.2 and 1.5 cM, respectively (Figure 3). The genomic DNAs of resistant and susceptible F_2_ plants from crosses of CH7086 with susceptible lines CH5241 and Taichung 29, as well as the parents and resistant (B_R_) and susceptible (B_S_) bulks were tested for the presence of markers linked to *PmCH86*. The specific *PmCH86*-associated 500-bp band amplified by BQ246670 was inherited as a dominant marker (Table 4, Figure 4). Marker BQ246670 may be useful for marker assisted selection (MAS) and for pyramiding *PmCH86* with other powdery mildew resistance genes in wheat depending on marker polymorphisms.

### Comparison of *PmCH86* and other *Pm* genes on 2BL

*Pm6* was resistant only to race E15, and susceptible to races E1-3, E5-7, E10, E13, E16-18, E20-21, E23, E26, E30-32, and E42 at the seedling stage [16]. Timgalen carrying *Pm6* was highly susceptible (IT 3-4) to E21 and E26, whereas CH7086 was highly resistant (IT 0-0;) (Table 1). This indicated that *PmCH86* differs in specificity from *Pm6*.

In order to further clarify the genetic relationship of *PmCH86* and *Pm6*, 236 F_2_ plants from CH7086/Timgalen were inoculated with E15, an isolate avirulent to both parents. Two susceptible plants were found, confirming that *PmCH86* and *Pm6* were not allelic, but were also not genetically independent (χ^2^_15:1_ = 11.76, *P*_df1_ = 0.001<0.01).

## Discussion

Alien gene transfer has an important role in increasing the genetic diversity available for wheat improvement [4]. *Th. ponticum* is immune to wheat powdery mildew and certain wheat-*Th. ponticum* derivatives are highly resistant to Chinese *Bgt* isolates. A resistance gene was recently found in two *Th. ponticum*-derived partial amphiploids [26]. However, there is no published report of transfer of powdery mildew resistance from this species to a wheat chromosome. In this study, CH7086 was produced by crossing and backcrossing Xiaoyan 7430 with susceptible wheat cultivars and selecting for powdery mildew resistance. A novel powdery mildew resistance gene, presumably transferred from *Th. ponticum* into common wheat, was mapped on chromosome arm 2BL and closely linked SSR markers were identified. However, based on GISH and Giemsa-C banding analyses of CH7086, no cytological evidence was found for an alien translocation. The gene *PmCH86* must be either present in a cryptic translocation involving a small chromosome segment from *Th. ponticum*, or a wheat gene derived from an unknown source. Cryptic alien transfers have been reported in other studies [6],[7]. Further studies are needed to determine the source of *PmCH86*.

Fivepowdery mildew resistance genes, *Pm6*, *Pm26*, *MlZec1*, *Pm33*, and *MlLX9*, were previously located on chromosome arm 2BL. *Pm6* originated from the G genome of *T. timopheevii* and *Pm33* was identified in *T. carthlicum* [27],[23]. *Pm6* and its linked RFLP marker *Xbcd135* were physically located at the region of deletion bin 2BL-6 (FL 0.89–1.00) [28], and this marker was subsequently converted into two STS markers, STSBCD135-1 and STSBCD135-2, which are closely linked to *Pm6* with a genetic distance of 0.8 cM [27]. Recently, Qin et al. developed a high-density genetic linkage map of the *Pm6* locus through a comparative genomics analysis using the genome sequences of rice and *Brachypodium* together with Triticeae ESTs, and localized *Pm6* at 0.2 to 1.2 cM proximal to the *Xbcd135* locus. However, in the present study, *PmCH86* was 8.2 cM distal to *Xbcd135* (Figure 3). The low infection type conferred by *PmCH86* to all races tested (Table 1) was also different from that of Timgalen [16].

Based on a microsatellite map, *T. carthlicum*-derived powdery mildew resistance gene *Pm33* was placed in the region between 18.1 cM distal to *Xgwm526* and 1.1 cM proximal to *Xwmc317* on chromosome 2BL, and the genetic distance between *Pm33* and *Pm6* was estimated to be 61.7 cM [23]. In our study, *PmCH86* was placed between *Xwmc332* and *Xwmc317* with an estimated genetic distance being about 30 cM proximal to *Xwmc317* (Figure 3). This indicates that *PmCH86* should not be allelic to *Pm33*. Yin et al. [24] located *PmJM22* on 2BL in wheat cultivar Jimai 22 using microsatellite markers. Since *PmJM22* was close to the region of *Pm33*, they may be allelic. *MlZec1*, a dominant resistance gene derived from wild emmer, was mapped distally to SSR marker *Xwmc356* on the terminal bin 2BL 0.89–1.00 [29]. *Xwmc356* was distal to *Xwmc317* [22], thus being at a different position from*PmCH86*. The recently identified gene *MlLX99* was derived from commercial winter wheat cultivars Liangxing 99. *MlLX99* was located on chromosome 2BL in the deletion bin 2BLl2-0.36-0.50 and was linked to SSR marker *Xgwm120* [30]. Based on these results and our own data, it appears that *PmCH86* is different from other known powdery mildew resistance genes on chromosome 2BL and represents a new powdery mildew resistance locus, and was therefore designated as *Pm51*. The genetic map of 2BL presented here is in minor conflict with *PmJM22* at map distance of BE405017 and BE444894 on the previously published map by Yin et al. [24]. This is likely due to our map being significantly longer. Similar phenomena were also hypothesised for small putative segmental introgressions carrying stem rust resistance from *Ae. speltoides* into wheat [31].

The wheat chromosome arm 2BL appears to be a hotspot region in disease resistance genes. Rust resistance genes, *Yr5*, *Yr7*, *Yr43*, *Yr44* [32],[33], *Lr48*, and *Sr28* [34],[35], have been mapped by molecular markers. Genes *Sr9* (several alleles) and *Sr16* were also placed in this arm [36]. The accumulation of functional markers in 2BL can be used to target resistance genes in the 2BL terminal regions. Comparative maps will be useful for isolating *Pm51* from a gene-rich region in the terminal region of wheat chromosome 2BL by using the rice and *Brachypodium* genomes as references. Saturation mapping of *Pm51* with more functional markers is underway to better understand the allelic relationships, gene structure and function in this gene-rich region.

We found that the new powdery mildew resistance gene *Pm51* mapped distally on chromosome 2BL was close to the EST-PCR marker BQ246670 (1.5 cM distal). Tightly linked markers for the *Pm51* locus characterized in this study could be used for MAS of *Pm51* in wheat breeding programs or to pyramid multiple resistance genes in a single genotype in order to achieve more durable resistance.

## Supporting Information

Table S1 **Molecular markers mapped to the *PmCH86* region based on wheat, rice, and *Brachypodium* synteny.**
(DOCX)
