# Supplementary material for: Chromosomal Location and Comparative Genomics Analysis of Powdery Mildew Resistance Gene Pm51 in a Putative Wheat-Thinopyrum ponticum Introgression Line
Source: PLoS One. 2014 Nov 21;9(11):e113455. doi: 10.1371/journal.pone.0113455 (PMC4240596; doi:10.1371/journal.pone.0113455)
Supplement: Table S1 — Molecular markers mapped to the PmCH86 region based on wheat, rice, and Brachypodium synteny. (DOCX) [file pone.0113455.s001.docx]

| **Table S1.** Molecular markers mapped to the *PmCH86* region based on wheat, rice, and *Brachypodium* | | | | |
| --- | --- | --- | --- | --- |
| orthologous | | | | |
| Marker | EST | Rice region | *Brachypodium* region | Wheat Bin |
| *Cos66* | CINAU140 | LOC_Os04g54870 | Bradi5g23570 | 2BL6-0.89-1.00 |
| *Xbcd135* | BE438862 | LOC_Os04g56350 | Bradi5g24660 | 2BL6-0.89-1.00 |
| *Cos55* | BQ169948 | LOC_Os04g56740 | Bradi5g25090 | 2BL6-0.89-1.00 |
| BI479701 | BI479701 | LOC_Os04g55480 | Bradi5g23970 | 2BL6-0.89-1.00 |
| P79 | BQ169948 | LOC_Os04g56740 | Bradi5g25090 | 2BL6-0.89-1.00 |
| BQ246670 | BQ246670 | LOC_Os04g57140 | Bradi5g25390 | 2BL6-0.89-1.00 |
| BE444894 | BE444894 | LOC_Os04g57440 | Bradi5g25637 | 2BL6-0.89-1.00 |
| BE405017 | BE405017 | LOC_Os04g57560 | Bradi5g25740 | 2BL6-0.89-1.00 |
